# The complete chloroplast genome of *Berchemia lineata*, an important medicinal plant from China

**DOI:** 10.1080/23802359.2020.1791749

**Published:** 2020-07-20

**Authors:** Xiaohong Xie, Detuan Liu, Hafiz Muhammad Wariss

**Affiliations:** a College of Design Art and Architectural Sciences, Zhejiang Wanli University, Ningbo, China; b Yunnan Key Laboratory for Integrative Conservation of Plant Species with Extremely Small Populations, Kunming Institute of Botany, Chinese Academy of Sciences, Kunming, China; c Key Laboratory for Plant Diversity and Biogeography of East Asia, Kunming Institute of Botany, Chinese Academy of Sciences, Kunming, China

**Keywords:** *Berchemia lineata*, medicinal plant, chloroplast genome, phylogenetic analysis

## Abstract

*Brechemia lineata* is a well-known medicinal plant in the Rhamnaceae family and widely used in traditional Chinese medicine. Here, we sequenced the complete chloroplast genome using Illumina pair-end sequencing data. The chloroplast genome was 154,962 bp in length, consisting of a large single-copy (LSC) region of 82,928 bp, a small single-copy (SSC) region of 17,376 bp, and a pair of inverted repeat (IR) regions of 27,329 bp. The chloroplast genome consists of 112 unique genes, including 78 protein-coding genes, 30 transfer RNA, and 4 ribosomal RNA genes. The overall GC content of the chloroplast genome was 37.0%. The phylogenetic analysis suggests close relationship of *B. lineata* with other *Berchemia* species. These genomic resources will be valuable resource for systematic and phylogenetic studies of *Berchemia* genus.

*Berchemia* is a genus of Rhamnaceae family, comprises of about 32 species and mainly distributed in temperate and tropical areas of East to Southeast Asia (Chen and Carsten 2007). *Berchemia lineata* (L.) DC. is a traditional Chinese medicinal species distributed in China (Fujian, Guangdong, Guangxi and Hainan), Japan and Vietnam. *Berchemia lineata* roots and leaves are used medicinally for relieving coughs and reducing sputum and for treating injuries, trauma, and snakebites (Chen and Carsten 2007). *Berchemia* species are often misidentified due to extremely similar vegetative morphological characteristics (Guo et al. 2016). Chloroplast genome has been broadly used for reconstructing phylogenetic relationships and development of molecular maker for the identification of plant species (Jansen et al. 2007; Huang et al. 2020). In this study, we sequenced the chloroplast genome of *B. lineata* to provide valuable genomic resources to facilitate systematic and phylogenetic studies of this important medicinal plant.

*Berchemia lineata* fresh leaves were collected from Zengcheng district in Guangzhou city (Guangdong, China: N23°13′49.0″, E113°45′45.2″) and voucher specimen (Zhou2019-18) was deposited in the Herbarium of Sun Yat-sen University (SYS). Genomic DNA was isolated using Tiangen plant genomic DNA kits (Tiangen Biotech, Beijing) and sequenced on the Illumina Hi-Seq 2500 platform. GetOrganelle pipeline were used for *de novo* chloroplast genome assembly (Jin et al. 2019). The chloroplast genome was annotated using Geseq and then manually verified and visualized in Geneious Prime v.2019.1.3 (Kearse et al. 2012; Tillich et al. 2017). Finally, circular chloroplast genome map was drawn using OGDRAW (Lohse et al. 2013). The chloroplast genome was deposited in the GenBank with the accession number MT621210.

*Berchemia lineata* chloroplast genome had quadripartite structure with 154,962 bp in length, consisted of a large single-copy (LSC) region of 82,928 bp, a small single-copy (SSC) region of 17,376 bp, and a pair of inverted repeat (IR) regions of 27,329 bp. The chloroplast genome contained 112 unique genes, including 78 protein-coding genes, 30 transfer RNA genes, and 4 ribosomal RNA genes. The overall GC content of the chloroplast genome was 37.0% and the corresponding values of LSC, SSC, and IR regions were 34.7%, 31.2%, and 42.4%, respectively.

Phylogenetic relationship of *B. lineata* within the Rhamnaceae family was inferred using the previously published eight chloroplast genome from the Rhamnaceae family and *Hippophae rhamnoides* and *Elaeagnus macrophylla* (Elaeagnaceae) as out group. The chloroplast genome of these species were aligned using MAFFT v7.3 (Katoh and Standley 2013) and MEGA7 were used to construct neighbor-joining phylogenetic tree (Kumar et al. 2016). Phylogenetic analysis revealed that *B. lineata* clustered with *Berchemia* species, however making a separate clade (Figure 1) might be due to geographical isolation from *B. berchemiifolia* (only distributed in the Korean peninsula and Japan) (Cheon et al. 2018). This chloroplast genome of *B. lineata* will provide a fundamental resource for studying the systematic position and conservation of this important medicinal species.

**Figure 1.**
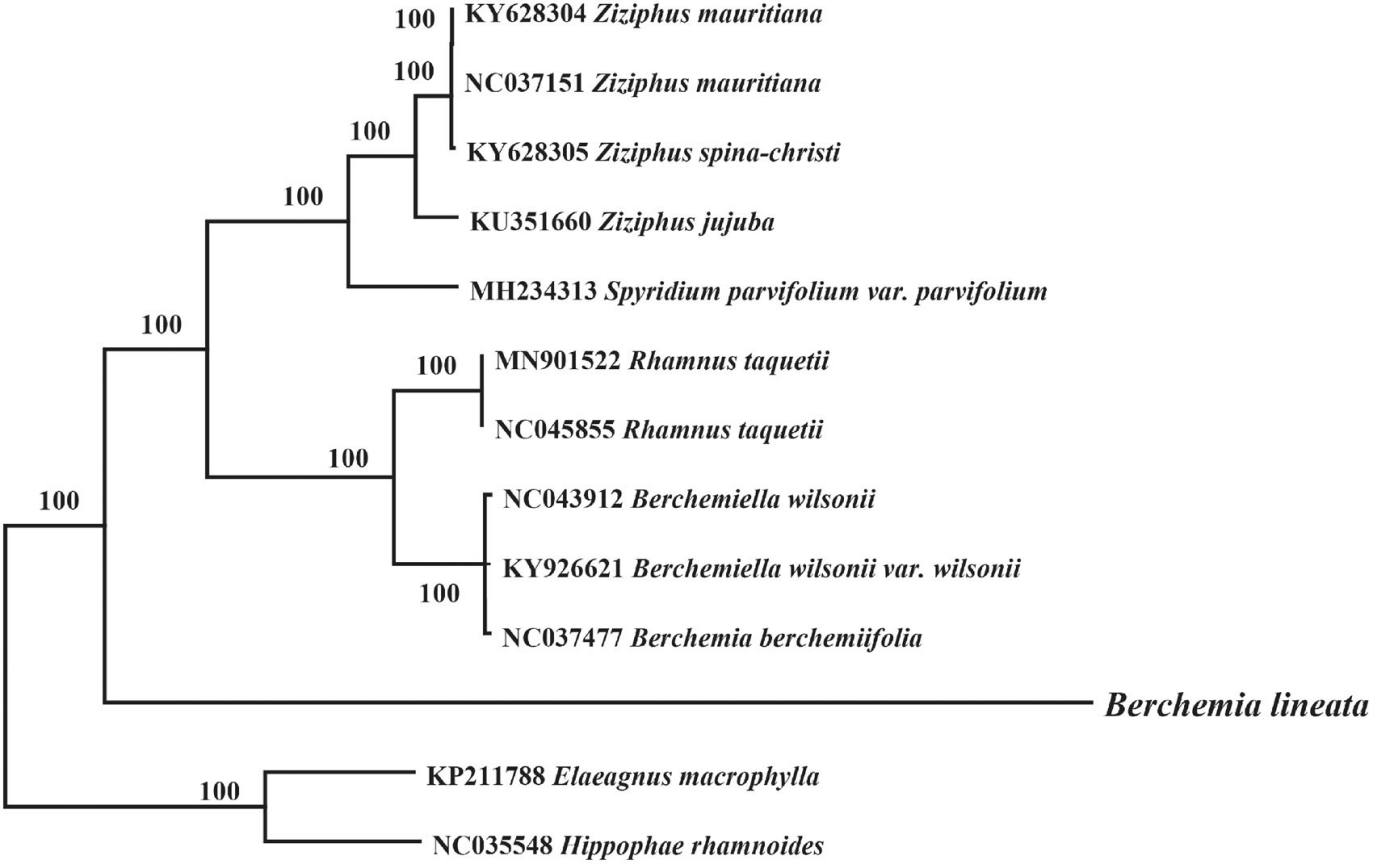
The phylogenetic tree based on 13 complete chloroplast genome sequences. The number on each node indicates bootstrap support value. The NCBI accession number for each species is given before its scientific name.

## Data Availability

The data that support the findings of this study are openly available in NCBI GenBank at https://www.ncbi.nlm.nih.gov/, reference number MT621210.

## References

[CIT0001] Chen Y, Carsten S. 2007. Rhamnaceae. Flora of China. Beijing: Science Press.

[CIT0002] Cheon KS, Kim KA, Yoo KO. 2018. The complete chloroplast genome sequence of *Berchemia berchemiifolia* (Rhamnaceae). Mitochondrial DNA Part B. 3(1):133–134. 33474094 10.1080/23802359.2018.1431068 PMC7800589

[CIT0003] Guo LC, Zhao MM, Sun W, Teng HL, Huang BS, Zhao XP. 2016. Differentiation of the Chinese minority medicinal plant genus *Berchemia* spp. by evaluating three candidate barcodes. Springerplus. 5(1):658–658. 27347459 10.1186/s40064-016-2207-4 PMC4899350

[CIT0004] Huang R, Liang Q, Wang Y, Yang TJ, Zhang Y. 2020. The complete chloroplast genome of *Epimedium pubescens* Maxim. (Berberidaceae), a traditional Chinese medicine herb. Mitochondrial DNA Part B. 5(3):2042–2044. 33457735 10.1080/23802359.2020.1756490 PMC7781925

[CIT0005] Jansen RK, Cai ZQ, Raubeson LA, Daniell H, Leebens-Mack J, Muller KF, Guisinger-Bellian M, Haberle RC, Hansen AK, Chumley TW, et al. 2007. Analysis of 81 genes from 64 plastid genomes resolves relationships in angiosperms and identifies genome-scale evolutionary patterns. Proc Natl Acad Sci USA. 104(49):19369–19374. 18048330 10.1073/pnas.0709121104 PMC2148296

[CIT0006] Jin JJ, Yu WB, Yang JB, Song Y, dePamphilis CW, Yi TS, Li DZ. 2019. GetOrganelle: a fast and versatile toolkit for accurate de novo assembly of organelle genomes. bioRxiv 4:256479. 10.1186/s13059-020-02154-5 PMC7488116 32912315

[CIT0007] Katoh K, Standley DM. 2013. MAFFT multiple sequence alignment software version 7: improvements in performance and usability. Mol Biol Evol. 30(4):772–780. 23329690 10.1093/molbev/mst010 PMC3603318

[CIT0008] Kearse M, Moir R, Wilson A, Stones-Havas S, Cheung M, Sturrock S, Buxton S, Cooper A, Markowitz S, Duran C, et al. 2012. Geneious Basic: an integrated and extendable desktop software platform for the organization and analysis of sequence data. Bioinformatics. 28(12):1647–1649. 22543367 10.1093/bioinformatics/bts199 PMC3371832

[CIT0009] Kumar S, Stecher G, Tamura K. 2016. MEGA7: molecular evolutionary genetics analysis version 7.0 for bigger datasets. Mol Biol Evol. 33(7):1870–1874. 27004904 10.1093/molbev/msw054 PMC8210823

[CIT0010] Lohse M, Drechsel O, Kahlau S, Bock R. 2013. OrganellarGenomeDRAW – a suite of tools for generating physical maps of plastid and mitochondrial genomes and visualizing expression data sets. Nucleic Acids Res. 41(Web Server issue):W575–W581. 23609545 10.1093/nar/gkt289 PMC3692101

[CIT0011] Tillich M, Lehwark P, Pellizzer T, Ulbricht-Jones ES, Fischer A, Bock R, Greiner S. 2017. GeSeq – versatile and accurate annotation of organelle genomes . Nucleic Acids Res. 45(W1):W6–W11. 28486635 10.1093/nar/gkx391 PMC5570176

